# Acceptance, Hesitancy, and Refusal in Anti-COVID-19 Vaccination: A Cluster Analysis Aiming at the Typology behind These Three Concepts

**DOI:** 10.3390/vaccines10091496

**Published:** 2022-09-08

**Authors:** Darie Cristea, Dragoș-Georgian Ilie, Claudia Constantinescu, Valeriu Fîrțală

**Affiliations:** Faculty of Sociology and Social Work, University of Bucharest, 010181 Bucharest, Romania

**Keywords:** COVID-19 vaccination, cluster analysis, behavior change, vaccine attitude, vaccine hesitancy, social norms, vaccination campaign

## Abstract

This paper presents the findings of a study aiming at an innovative typology of attitudes towards COVID-19 vaccination. The proposed typology tries to go beyond the common sociological studies who divide the public into three categories: pro-vaxxers, anti-vaxxers, and hesitants. Our purpose is obtaining a more complex typology using cluster analysis. The article is based on a nation-wide survey conducted in Romania. The sample of the survey was statistically representative to the population of Romania and was composed of 1002 participants. A k-means algorithm for classifying cases was used to identify how the studied population structures itself when it comes to attitudes towards COVID-19 vaccination. We see hesitancy as an expression of concern or doubt about the value or safety of the vaccination, but also as fear or dis(trust) in the authorities, or as disinterest. We found out that the Romanian public falls into three categories regarding the attitude towards COVID-19 vaccination: the “non-fanatical” pro-vaxxers, the anti-vaxxers, and those without a clear opinion (uninterested and/or undecided). What we usually call “vaccine hesitancy” can be found, depending on motivation or type of hesitancy, in both of the last two clusters.

## 1. Introduction

Vaccination (and the attitude towards vaccination) is a major topic at the intersection of medical and social sciences even before 2020. After the emergence of the COVID-19 pandemic, it is clear that the issue of vaccination acceptance is more complex and intricate than envisaged.

In the past two years of COVID-19, countless articles were published on accepting and refusing vaccination, especially in the context of the pandemic. We are far from benefiting from all the information these studies contain. There is so much information that it is practically impossible to assimilate. It would be necessary to collect the most relevant findings in order to clarify the identified typologies and causalities.

To the extent of our documentation, we observe that there are several key concepts, based on empirical and measurable results: acceptance of vaccine, confidence in the vaccine, hesitation towards the vaccine, and refusal of the vaccine [1,2,3,4,5].

An important problem to solve is whether we are dealing with a continuum phenomenon or with separate phenomena. It is a problem of scaling, common in the social sciences. In general, the attitude or intention of vaccination is measured in opinion polls with an ordinal question of acceptance/indecision/refusal type, where, sometimes, the indecision is detailed in 2–3 other variants of answer regarding the motivations. But what if the acceptance and refusal of vaccination are not steps on a scale? What if they are qualitatively different phenomena, with motivations coming from different spectra and attributable to clearly differentiated human typologies?

The pandemic has also shown us that the triangle of acceptance/hesitation/refusal has a special dynamic, depending on the availability of the vaccine, what rumors/fake-news appear around COVID-19 and the vaccine, trust in the authorities, fear, perception of the social norm, restrictions on the unvaccinated, etc. In rural areas, the dynamics of this triangle can also be influenced by the population’s access to health services, and the development of Multifunctional Integrated Community Centers could be useful in these specific areas [6].

The pandemic context determined in many situations the association of vaccination with the intention to avoid restrictions imposed by the authorities on the unvaccinated. Thus, a non-medical motivation for administering the vaccine arose: to avoid restrictions. Or, on the contrary, the refusal of the vaccine emerged as a form of social resistance to too high regulatory pressure from the authorities. Such type of paradoxical social polarization raises serious questions about the most effective method of regulation implemented by the authorities in any future crisis. In crisis situations, social discipline and compliance with norms are much more important than in other social and historical situations.

Studies show that there are multiple explanations on the acceptance/refusal of vaccination. The acceptance, hesitation, or refusal of vaccination (in the case of COVID-19 and beyond) have been explained by the following types of causal models:

1 Models related to vaccine mistrust (mistrust in the content of the serum or the production method), medical mistrust, and scientific illiteracy (although, the relationship between scientific literacy and the intention to vaccinate is much more complicated than it seems) [7,8].
2 Models that refer to the mistrust in the authorities, to conspiracy theories, and the assumption of hidden intentions regarding the organization of the vaccination process by the authorities [9,10,11,12,13].
3 Models that refer to differences in perception of vaccine and vaccination between different social demographic categories (age, education, gender, national membership, religion, etc.) [14,15,16].
4 Models that primarily relate to the channel, the source of information about the virus, the pandemic, the vaccine, and the vaccination process [17,18].
5 Psychosociological models, that consider purely psychosociological variables that are associated with acceptance or hesitation regarding vaccination—for example, in a previous article we argued that there is a very strong correlation between positive attitudes towards vaccination and the belief that most others want to get vaccinated [19].

The question is if the typology based on the triangle acceptance/hesitation/refusal is too obvious and too simplistic. If these three attitudes were described by more pragmatic indicators, a much more operational typology would prevail. This would also show us what are the resorts of this division, according to the attitude towards vaccination, a division that has already acquired social political valences in the last year.

Therefore, our research question is the following: could we get a more explicit typology of the attitude towards vaccination in Romania if, instead of the direct question on the intention to vaccinate, we would use a set of questions that would break down on several dimensions the acceptance/hesitation/refusal triplet in the case of COVID-19 vaccination? Such a typology may show us the resorts underlying this public opinion/attitude towards the COVID-19 vaccination.

We intend to build this typology through k-means clustering. Details about the data used are available in the Materials and Methods section. First, a brief review of three studies of this type published recently on the anti-COVID-19 vaccination. One is based on data collected in the U.S., two others relate to the case of Europe and Romania in particular, our study being based on data from Romania. 

A survey conducted in 2021 on the acceptance and hesitation of vaccines among 2491 healthcare workers in Southern California aims to demonstrate that there is a heterogeneous group (and subgroups) with a different attitude toward vaccination, not just the “anti” and “pro” vaccine. The clustering analysis conducted by the authors of this study starts from the idea of a continuum between total acceptance and complete refusal and describes groups and subgroups of healthcare workers who hold varying degrees of indecision about vaccination.

Respondents to the study were grouped into four clusters: (1) misinformed, (2) uninformed, (3) undecided, and (4) unconcerned. Thus, there is diversity in vaccine hesitancy and their conclusions are that “messaging should be adapted to specific subgroups to increase understanding of the science behind vaccine” [20].

Vulpe and Rughiniș, using the Eurobarometer 91.2 survey conducted between 15 and 29 March 2019, identified three configurations of persuasion regarding the effectiveness, safety, and usefulness of the vaccine: hesitant, confident, and trade-off.

The authors’ conclusion when conducting a cluster analysis includes referring to substantial variation at the country level, but they cannot find “strong social demographic differences between the three clusters of beliefs”.

One of the findings of this study is the “need to address the socially amplified risk of likely vaccine damage and to consider compromise models of concurrent trust and distrust in vaccines evaluation” [21].

The study “Social worlds of attitude towards anti-COVID-19 vaccination: Romanians in the European context”, published in 2021, is based on the Flash Eurobarometer State of the European Union. The subsequent analysis of the clusters starts from the idea that the attitude towards the vaccine is not only quantitative between the two surveys: pro- and anti-vaccine. The cluster analysis combines three indicators and generates six types of vaccine attitudes. Romania is positioned in the European context in terms of specific attitude profile alongside Poland, the Czech Republic, and Lithuania.

An important finding of the author is that we have interposing qualitatively structured categories between the pro-vaccine, anti-vaccine, or hesitant segments of the public. It seems to the researcher that both vaccination attitudes and vaccination intentions depend on the social demographic elements and previous life experiences. The conclusion of this study is that we are dealing with “social worlds of the attitude towards COVID-19 vaccine in the language communities on this issue” and these social worlds are structured differently in groups of countries. A more accurate understanding can be obtained through additional studies. These studies should use variables such as the stage of SARS-CoV-2 infection, migration experiences, and the feeling of belonging to national/regional spaces with different cultural models [22]. 

## 2. Materials and Methods

The data used for this analysis comes from a larger public opinion survey, conducted on a nationally representative sample for Romania. Two of the co-authors of this article were decisively involved in conducting the survey (see note [23], explaining the full context of the survey and providing all the data necessary to identify it, including the research report). The data was collected between 1 and 10 October 2021, by LARICS, a public opinion analysis laboratory known in Romania, under the auspices of the Institute of Political Science and International Relations within the Romanian Academy.

Therefore, the analysis was carried out on a database of 1002 respondents in the territory of Romania; the sample was statistically representative at the level of the entire non-institutionalized population in Romania, aged between 18 and +65 years. It was a multilayer probabilistic sample, and the margin of error was 3.1%. The questionnaire was applied by phone using CATI as a method of coordinating interviews. The sample was validated based on the data available at the National Institute of Statistics in Romania.

To identify how the studied population structures itself when it comes to attitudes towards vaccination, we decided to use an algorithm for classifying cases. The cluster is a group of subjects or objects that have common traits and are grouped together based on this similarity [24]. The components of the cluster are similar to each other but are different from the elements that set up another cluster [25]. To study the distribution of cases in clusters we decided to use the “k-means” clustering algorithm method. This procedure consists of measuring the proximity of cases to the average of a cluster. “K” is the number of clusters in an analysis that is chosen by the researcher. The “k-means” algorithm proposed by J. MacQueen assigns a random average for each cluster (for example, for 3 clusters we will have 3 averages). Then, it measures the proximity of the observed cases to this initial average, after which it calculates the final average of the cases in the cluster and groups them within these clusters [26]. The belonging of an observed case to the cluster is determined by the proximity of the observed value to the average value “k” of the cluster.

Using the IBM SPSS 2.0 statistical analysis program, we conducted a series of tests in our database, including, most importantly, “k-means” cluster analysis. Our analysis resulted in several tables, for models with 2, 3, 4, and 5 clusters.

## 3. Results

Tests in SPSS were conducted with a distribution in 2, 3, 4, and 5 clusters. While analyzing this distribution of cases in clusters, we found that a distribution of cases in 3 clusters (see Table 1) represents an unfragmented classification of the population, in other words, the studied population tends to group around these average values of the clusters, and we can observe the significant differences between them. 

The clustering was made around 6 statements. The statements measured scalarly, on a scale of 5 steps, the anchor to: (i) confidence in the safety of vaccination against COVID-19; (ii) confidence that vaccination against COVID-19 is abusively promoted by the state authorities; (iii) the belief that vaccination against COVID-19 is the only way the pandemic can be stopped; (iv) the fact that the pandemic does not exist or is an exaggeration, (v) the fact that vaccination against COVID-19 is not effective; and (vi) the fact that vaccination against COVID-19 should be mandatory. The lower the value in the table, the lower the agreement/confidence level.

The clusters have the following composition in terms of the number of subjects:

- Cluster anti-vaccination subjects (A): 378 subjects.
- Cluster followers of vaccination (B): 356 subjects.
- Cluster those without an opinion (C): 260 subjects.

The result is 994 subjects who answered this set of questions, out of a total of 1002 participants, 8 subjects refusing to answer these questions.

In order to identify the significant differences that occur between the members of the 3 clusters we decided to apply Fischer′s "z-test", the results of which may be seen in Table 2.

The subjects were analyzed comparatively according to the following variables: age, gender, residence environment, income, educational level, from where they tend to get informed about the pandemic situation, and how the family doctor advised them whether to get vaccinated or not.

In the table above, we analyze the statistically significant differences between the values on the columns. We observe that the members of the first cluster (A) are quite young, tend to live in urban areas, have an average or high level of income, have an average or higher education, tend to be male, to inform themselves about vaccination and the pandemic from official sources, social media or the internet, and tend either to not have talked to the family doctor about the vaccination, or to declare that the family doctor has advised them not to get vaccinated against COVID-19.

The subjects in cluster B tend to be quite old, rather urban, have an average or high income, have either primary education or higher education, be female, obtain information from TV, from family, or official websites about the pandemic, and it is the group that seems to have been advised to be vaccinated against COVID-19 by the family doctor.

The subjects in cluster C tend to be adults or old, tend to come from rural areas, tend to have low incomes, primary or secondary education, tend to obtain information from TV, and the family doctor has advised them either not to get vaccinated against COVID-19 or to wait.

## 4. Discussion

We recall that the result of the cluster analysis is presented in Table 1. We also recall that the values describing the clusters move between 1 and 5, 1 indicating total disagreement with the respective statement, and 5 a total agreement. The middle value is 3. As already mentioned, we chose a typology with three categories.

Cluster A is composed of anti-vaccination subjects: they do not believe that COVID-19 vaccination is safe, nor is it the only method that can stop the pandemic; they believe that vaccination is abusively promoted by the authorities and is not effective. They consider the pandemic a lie/exaggeration and strongly oppose a possible mandatory vaccination.

Cluster B is the followers of vaccination’s cluster. We notice from the beginning that this cluster is much more nuanced and less firm in its positions than the anti-vaxxers’ cluster. The subjects in cluster B are very convinced that vaccination is safe and believe (not as strongly) that vaccination is the only measure to stop the pandemic. Obviously, they are not conspirators (they do not believe the pandemic is a lie) and they do not think that COVID-19 vaccination is not effective. On the other hand, they do not have a firm opinion on mandatory vaccination, and they neither agree nor disagree with the idea that vaccination is a measure abusively promoted by the authorities (although, as pro-vaxxers, they would have been expected to disagree with this idea).

Cluster C has almost all the coefficients at the middle of the range (value: 3), which indicates not so much a cluster of hesitants, but rather of people who have not formed an opinion—those without opinion (they are rather undecided or, perhaps, uninterested). The only element at which the coefficient 3 is not obtained is the first (the idea that the vaccine is safe) and there, the coefficient 2 indicates a slight disagreement, so a slight fear about the safety of the vaccine. In this cluster there are significantly fewer cases than in the other two.

Usually, when opinion polls measure vaccination attitudes or vaccination intent, they work with categories such as: acceptance/hesitation/refusal or strong acceptance/easy acceptance/easy refusal/strong refusal. Below you may see how our clusters can change the optics on this typology.

First, we are focusing our discussion not on a mere linear attitude towards COVID-19 vaccination, but on the attitude of our subjects towards the COVID-19 pandemic, on how the government has managed this crisis, and how the subject has viewed the pandemic. Our findings show that young people in Romania tend to form the ‘antivaccination subjects’ cluster (A), adults being the ones that tended to group in the ‘followers of vaccination’ cluster (B) and older subjects tended to group in the cluster of ‘those without opinion’ (C). 

Opposite of what was expected, the members of cluster A tended to be more educated than the members of cluster C. They also tend to live in urban areas, to be male, and to have higher incomes than the members of cluster C. The cluster ‘followers of vaccination’ tends to be composed more of elderly women with higher education living in urban areas. This could show that education, the residency environment, and income are not predictors of vaccine acceptance or hesitation in Romania.

As seen in Table 2, based on the structure of the clusters, and as we argued above, the social demographic features are not a relevant predictor of vaccination attitude in Romania. Attitudes towards vaccination tend to be centered more on the individual’s subjective views regarding vaccine and vaccination. We believe that the failure of the vaccination campaign in Romania is based on the rather large size of clusters A and C, and their high incidence in the Romanian population. In total, 63% (638 subjects) of our entire sample could be classified as part of one of these two clusters. Less than 40% can be found in the pro-vaxxers cluster (B). This matches the fact that Romania had an official COVID-19 vaccination rate of about 40% at the time of our survey.

To create effective public-health policies for future pandemics, governments need to understand that clusters A and C are formed around distrust of the state, possibly misinformation, and a lack of understanding by the population of what a health crisis is. We can prevent future deaths from pathogens and future shutdowns of our medical systems by finding ways to reduce the size of clusters A and C and increase cluster B.

In order to observe the differences between the clusters in terms of attitude towards vaccination, we compared the cluster membership with the answers to the next question in our survey, “What do you think about the COVID-19 vaccine?”. As we show below, there are some significant differences between the three clusters when it comes to what their members think about COVID-19 vaccination.

In Table 3 we created a matrix that shows the affirmative answer to a set of claims regarding vaccination in general (other than anti-COVID-19 vaccine) and vaccination against COVID-19, within our three clusters.

As it can be observed from Table 3, antivaccine subjects tend not to have a problem with vaccination in general, but with anti-COVID-19. Members of this cluster tend to believe that it is not good to get the COVID-19 vaccine, therefore, they oppose mandatory vaccination. The problem seems to be not with vaccination in general, but with vaccination against COVID-19. Another interesting fact is that 49.1% of respondents who said they were not convinced of COVID-19 vaccination were in this cluster. This shows that the cluster is composed of a mixture of respondents who are either anti-vaccination against COVID-19 or are not convinced to get vaccinated in general.

The second cluster, followers of vaccination, has a strong positive attitude towards vaccination against COVID-19. Members of this cluster seem to believe that vaccination is good and all people should get vaccinated.

The third cluster, those without opinion, according to their name, do not tend to have a strong opinion on this topic, but only a high score on the question ‘I was not convinced’, as one might expect.

As seen in Table 4, there are significant differences between the attitudes of the members who make up the three clusters. Cluster A and cluster C tend to differ from cluster B in terms of belief in vaccination and in that they are not convinced to get vaccinated against COVID-19. Cluster B differs from the other two clusters in believing that the vaccine is good for humans and that it should be mandatory to vaccinate against COVID-19. However, even in cluster B, only 1/5 of the subjects agree with the mandatory vaccination.

## 5. Conclusions

To understand the attitude towards vaccination in times of crisis such as the COVID-19 pandemic, it is necessary to go beyond the simple categories: agreement, refusal, and hesitation. They play a crucial role in statistical description and even in forecasting public attitudes towards vaccination or a particular vaccine. A cluster analysis on relevant indicators can break down this typology into its essential attributes. Depending on the questions available and included in the analysis, clusters may be more or less relevant or may acquire new valences and highlight specific dimensions.

We argue that the indicators based on which we built the clusters, combined with the time of the survey (autumn of 2021), and with the fact that we had available data from a sociological survey that is nationally representative (for Romania), allowed us to perform a concise typology, which highlighted the dominant factors behind the acceptance/refusal/hesitation scheme.

Briefly, studies prior to the pandemic showed that Romanians were not significantly opposed to vaccination in general [27]. To the extent that they did not agree with vaccination, they were rather hesitant. This hesitation has many resorts: postponement, disinterest in the problem, waiting for others to vaccinate, waiting for clarifications or impositions from the authorities, etc. Managing hesitation and indecision is the secret to the success or failure of vaccination campaigns, perhaps more than managing refusal. At the end of 2021, strictly on the anti-COVID-19 vaccination, Romania was one of the most unvaccinated countries in the EU.

The studies quoted in the introductory section of the article show us that, from country to country, there is more than just acceptance, hesitation, or refusal behind the segmentation of the public according to the attitude towards the anti-COVID-19 vaccination [20,21,22]. Some of these attitudes can be dominated by information, others by disinterest, some by fear, others by (dis)trust in the authorities or by the belief that the problem will be solved until it is your turn to vaccinate. The agreement is not simply agreement, just as refusal is not simply refusal. And the segment of hesitants is a world in itself.

As a result, the main conclusions of our study are:

- Based on *the indicators considered in the analysis, we do not simply have an agreement/hesitation/refusal typology regarding the anti-COVID-19 vaccination in Romania. We have a cluster favorable to vaccination, one unfavorable, and one cluster disinterested in the vaccination issue rather than hesitant.*
- The anti-vaccine cluster is the most coherent: they do not believe in neither vaccination, nor the pandemic. They are also bothered by the promotion of vaccination by the authorities.
- The pro-vaccination cluster is not a fanatical one: the respondents trust that vaccination is safe and will solve the problem, but they do not necessarily trust the way the authorities promote it. The pro-vaccination cluster accepts mandatory vaccination, in comparison with the other two clusters. However, its respondents are not too excited about the idea, if we look at the score in the analysis (Table 1).

Briefly, our picture looks like this: we have a cluster of anti-vaxxers (over a third of the sample) that brings together traditional anti-vaxxers (those who oppose any vaccination), and the anti-vaxxers of the COVID-19 era (those who have shown this attitude in the context of the current pandemic). This cluster is strongly targeted against the vaccine, against public policy to promote vaccination, and to a certain extent it even denies the real size of the pandemic. The pro-vaccine cluster (about a third of the sample) believe the vaccine is both effective and safe. However, they do not believe vaccination should be imposed, and they are reluctant towards the way the state manages/communicates the pandemic. They are rather reasonable in relation to the sensitivities of others and they are not fanatical or intrusive. This attitude also explains to a great extent the apparent minority of the pro-vaccination speech in the Romanian public space during 2021. The third cluster, under a third of the public, is rather disinterested or without a clear opinion on vaccination and the pandemic. Vaccine hesitants, depending on motivation or type of hesitation, fall into both clusters A and C.

## Figures and Tables

**Table 1:** K-means cluster analysis; sample distribution by clusters (model with 3 clusters).

Respondent’s Opinion on COVID-19 and Vaccination	Cluster: Anti-vaccination Subjects (A)	Cluster: Followers of Vaccination (B)	Cluster: Those without Opinion (C)	Interquartile Range (IQR) 25%	Median Value 50% (IQR)	Interquartile Range (IQR) 75%
(A) Vaccination against COVID-19 is safe	2	5	2	1.00	3.00	4.00
(B) Vaccination against COVID-19 is a measure abusively promoted by the authorities	5	3	3	2.00	3.00	5.00
(C) Vaccination against COVID-19 is the only measure that can stop the pandemic	2	4	3	2.00	3.00	4.00
(D) This pandemic is more of a lie or an exaggeration	4	2	3	2.00	3.00	4.00
(E) Vaccination against COVID-19 is not effective	4	2	3	2.00	3.00	4.00
(F) Vaccination against COVID-19 should be mandatory	1	3	3	1.00	2.00	4.00

**Table 2:** Results of the z test (Fischer). The data from the table are statistically representative for the demographical structure of Romania in the year 2022. The sample data was collected randomly and is probabilistic with a ±3.1% error margin, on a sample of 1002 subjects from Romania.

Respondent’s Demographical Characteristics	Cluster: Anti-Vaccination Subjects (A) Results of the z Test	Size of Cluster A Row N % of Sample	(A) Number of Cluster Members	Cluster: Followers of Vaccination (B) Results of the z Test	Size of Cluster B Row N % of Sample	(B) Number of Cluster Members	Cluster: Those without Opinion (C) Results of the z Test	Size of Cluster C Row N % of Sample	(C) Number of Cluster Members	*p*-Value
Young (18–34 years old)	**B C**	59.4%	148		22.9%	57		17.7%	44	0.000
Adult (35–64 years old)		34.4%	178		34.9%	181	**A B**	30.7%	159	0.000
Old (+65 years old)		22.9%	52	**A C**	52.0%	118	**A**	25.1%	57	0.000
Urban	**C**	44.2%	253	**C**	37.5%	215		18.3%	105	0.000
Rural		29.7%	125		33.5%	141	**A B**	36.8%	155	0.000
Low income		31.0%	153		26.4%	130	**A B**	42.6%	210	0.000
Average income	**C**	44.9%	182	**C**	45.9%	186		9.1%	37	0.000
High income	**C**	41.5%	34	**C**	46.3%	38		12.2%	10	0.000
Low education		21.5%	32	**A**	47.0%	70	**A**	31.5%	47	0.000
Average education	**B**	39.7%	214		28.4%	153	**A B**	31.9%	172	0.000
High education	**C**	43.1%	132	**C**	43.5%	133		13.4%	41	0.000
(Obtains information about COVID-19 and vaccination from:) Friends		28.8%	156		36.0%	195		35.2%	191	0.000
(Obtains information about COVID-19 and vaccination from:) Facebook and the internet	**B C**	50.0%	11		31.8%	7		18.2%	4	0.000
(Obtains information about COVID-19 and vaccination from:) Family		50.5%	103	**C**	29.4%	60		20.1%	41	0.000
(Obtains information about COVID-19 and vaccination from:) Official gov. websites	**C**	25.8%	8	**C**	61.3%	19		12.9%	4	0.000
(Your family doctor) Advised you to get vaccinated against COVID-19		50.5%	94	**A C**	39.8%	74		9.7%	18	0.000
(Your family doctor) Advised you not to get vaccinated against COVID-19	**B**	66.7%	6		11.1%	1	**A B**	22.2%	2	0.000
(Your family doctor) Advised you to wait		28.2%	117		56.6%	235	**A B**	15.2%	63	0.000
(Your family doctor) You did not discuss the vaccine with your family doctor	**B C**	21.8%	19		4.6%	4		73.6%	64	0.000

**Table 3:** Answers of the cluster cases to the question measuring attitude towards vaccination.

Respondent’s Attitude towards COVID-19 Prevention Measures	Cluster: Anti-Vaccination Subjects (A)	Cluster: Followers of Vaccination (B)	Cluster: Those without Opinion (C)
I generally do not believe in vaccination	63.0%	3.9%	33.1%
I do not have a problem with vaccination in general, but I do not trust COVID-19 vaccination	61.1%	5.6%	33.3%
It has not convinced me, nor am I against it yet	49.1%	9.9%	40.9%
I think it is good to get vaccinated against COVID-19	14.6%	76.0%	9.3%
I believe that vaccination against COVID-19 should be mandatory for adults who do not have medical contraindications	5.3%	73.7%	21.1%
N/A	0.0%	66.7%	33.3%

**Table 4:** Results of the second z-test for differences between clusters to the question “What do you think about the COVID-19 vaccine?”.

Respondent’s Attitude towards COVID-19 Prevention Measures	Cluster: Anti-Vaccination Subjects (A)	Cluster: Followers of Vaccination (B)	Cluster: Those without Opinion (C)
I generally do not believe in vaccination	B		B
I do not have a problem with vaccination in general, but I do not trust COVID-19 vaccination	B		B
It has not convinced me, nor am I against it yet	B		B
I think it is good to get vaccinated against COVID-19		AC	
I believe that vaccination against COVID-19 should be mandatory for adults who do not have medical contraindications		AC	A

Results are based on double-sided tests with a significance level of 0.05. For each significant pair, the key category with the lower proportion of the column appears in the category with the higher proportion of the column.

## Data Availability

The results of the survey can be publicly viewed at this link: https://larics.ro/wp-content/uploads/2021/10/Barometru-Securitate_octombrie-2021-complet.pdf (accessed on 20 May 2022).
